# Rupture of a dissecting thoracoabdominal aortic aneurysm due to *Citrobacter freundii* infection

**DOI:** 10.1002/ccr3.4719

**Published:** 2021-08-30

**Authors:** Masafumi Hashimoto, Kenji Mogi, Manabu Sakurai, Tomoki Sakata, Kengo Tani, Yoshiharu Takahara

**Affiliations:** ^1^ Division of Cardiovascular Surgery Funabashi Municipal Medical Center Heart and Vascular Institute Funabashi Japan

**Keywords:** anatomical revascularization, *Citrobacter freundii*, omental flap wrapping, ruptured aortic aneurysm

## Abstract

We describe a case of an elderly man with *Citrobacter freundii*‐associated infectious rupture of a dissecting thoracoabdominal aortic aneurysm. We performed an emergency thoracoabdominal aortic replacement using a rifampicin‐soaked prosthetic graft and omental flap wrapping. The patient was discharged on postoperative day 255, although he experienced pseudomembranous enteritis and paraplegia.

## Graphical abstract

*Citrobacter freundii*, an opportunistic bacterium, can infect residual dissections in the descending aorta, and the resulting infectious aortic aneurysm could rupture in a week. We found that a rifampicin‐soaked prosthesis and omental flap wrapping were effective in preventing vascular prosthesis infection.

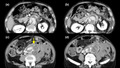

## 1 INTRODUCTION

Infectious aortic aneurysms are relatively rare and account for 0.7%–3.0% of all aortic aneurysms.^1^ As such, surgical management protocols for these cases are not well defined and postoperative infection control remains a concern.

Population‐based studies on ruptured thoracoabdominal aortic aneurysms (TAAA) have confirmed that less than half of the patients presenting with this condition survive until hospital evaluation.^2^ Operative mortality associated with open repair of ruptured TAAA ranges from 12% to 26%, but can be significantly higher when handled by less experienced surgeons.^3^ In a study, open repair of ruptured TAAA carried a mortality rate of 53%.^4^ Besides, it is known that infectious aneurysms caused by *Citrobacter freundii* ^5^ are rare.

Herein, we report a case of infectious rupture of a dissecting TAAA caused by *C*. *freundii*, which was successfully treated with anatomical revascularization using a rifampicin‐soaked prosthetic aortic graft and omental flap wrapping.

## 2 CASE PRESENTATION

This case report has been approved by the ethics committee of our institute. The patient provided informed consent for the publication of this report. The datasets generated during the current study are available from the corresponding author on reasonable request.

**FIGURE 1:**
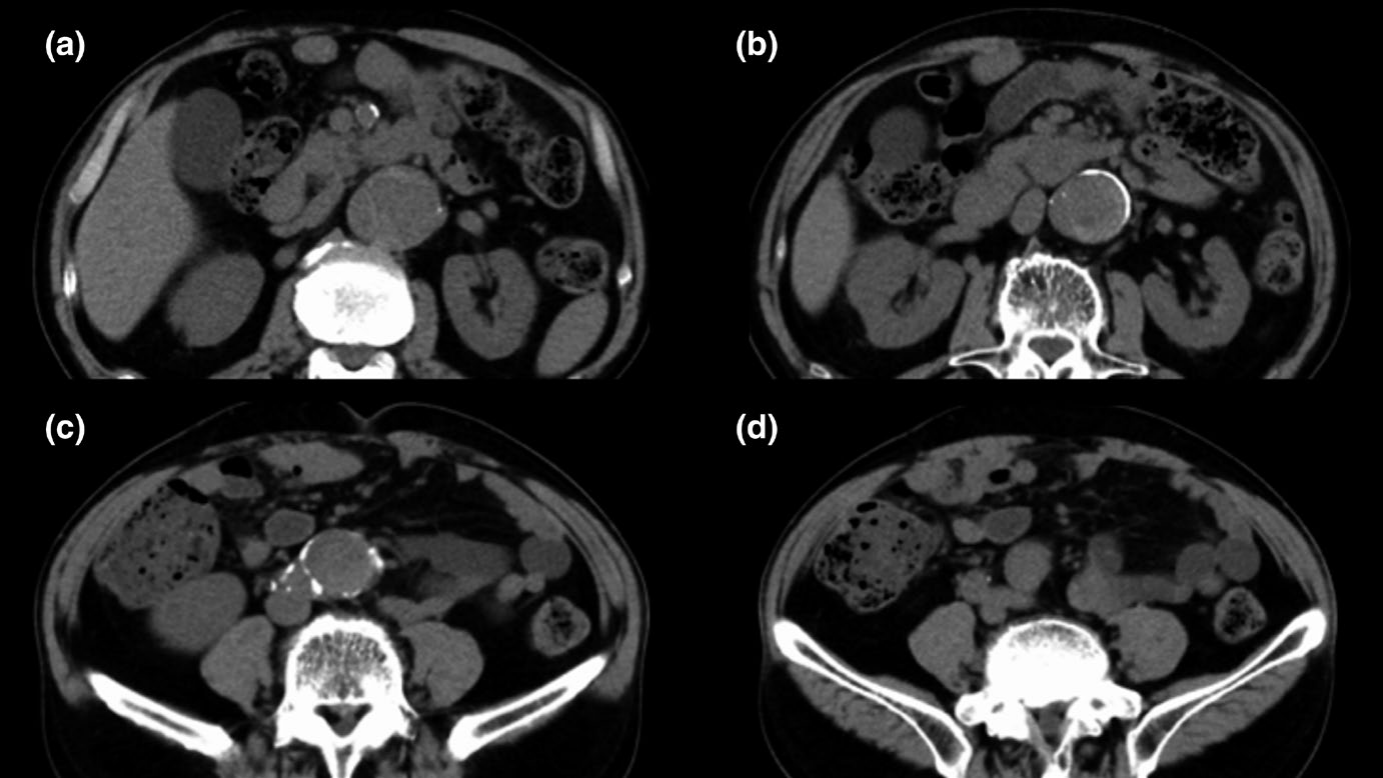
A computed tomography angiogram does not show aortic dilatation. The dissection in the thoracoabdominal aortic lesions has a maximum diameter of 38 mm. (A) At the level of the superior mesenteric artery, (B) at the level of the renal arteries, (C) infrarenal level, and (d) at the level of the terminal aorta

**FIGURE 2:**
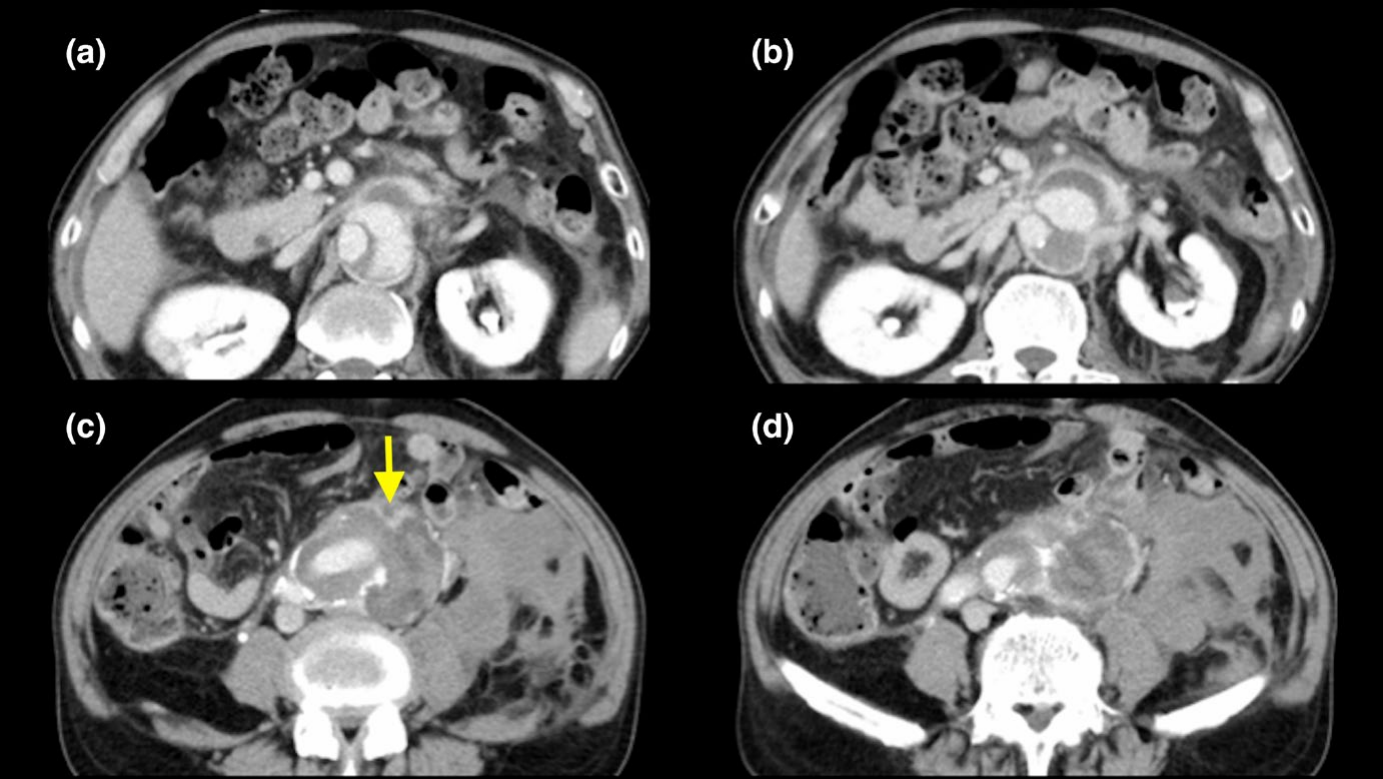
Computed tomography angiogram reveals a rupture of the adventitia (yellow arrow) and a hematoma in the retroperitoneal cavity. (A) At the level of the superior mesenteric artery, (B) at the level of the renal arteries, (C) infrarenal level, and (D) at the level of the terminal aorta. This computed tomography angiogram was obtained 1 week after the computed tomography angiogram (Figure 1) was taken

A 63‐year‐old man with complaints of fever that persisted for a week, sudden lower back pain, and hypotension was admitted to our hospital. On admission, he had a body temperature of 40℃, elevated white blood cell count (249×10^2^/μL), and C‐reactive protein level of 15.6 mg/dL. The patient had a history of hypertension and smoking, and had undergone replacement of the descending aorta for a dissecting aortic aneurysm 6 years ago. Computed tomography (CT) of the remaining thoracoabdominal aortic lesion showed no dilatation (Figure 1). The CT scan also revealed an acute rupture of the adventitia and a hematoma in the retroperitoneal space (Figure 2). He was diagnosed with infectious rupture of a dissecting TAAA, and emergency thoracoabdominal aortic replacement with omental flap wrapping was performed.

Dissection of the aorta just superior to the renal artery resulted in an abscess outflow into the abdominal cavity. Following complete excision of the aneurysm, the abdominal aorta and the renal artery were reconstructed using a rifampicin‐soaked Gelweave Coselli Thoracoabdominal Graft (Terumo Corporation, Tokyo, Japan). The proximal side of the dissected aorta was anastomosed to the prosthetic aortic graft from the previously replaced descending aorta. Since the aortic dissection extended to both iliac arteries, the abdominal aorta was also replaced using a Gelsoft Bifurcated Graft (Terumo Corporation, Tokyo, Japan). After weaning the patient from cardiopulmonary bypass, the omentum was passed into the retroperitoneal cavity through an incision in the ligament of Treitz and wrapped around the prosthetic aortic grafts.

*C*. *freundii* was detected in the intraoperative retroperitoneal abscess, as well as the preoperative and postoperative blood cultures. Similar bacteria were detected in the aortic aneurysm wall excised during surgery. The culture produced cephalosporinase (AmpC) and showed innate resistance to various penicillin antibiotics such as ampicillin and early‐generation cephalosporins.

**FIGURE 3:**
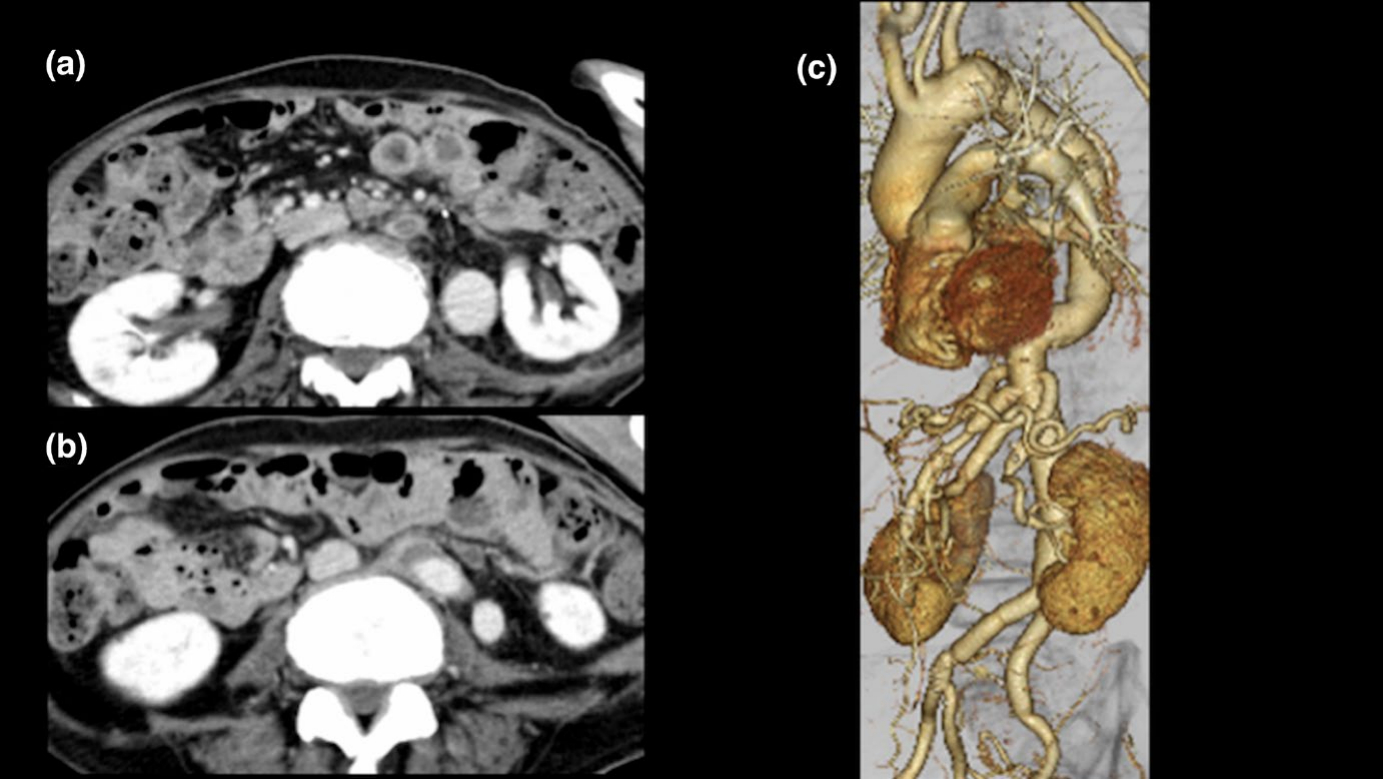
Postoperative computed tomography scan obtained at the time of discharge. (A, B) The scan shows the retroperitoneal space without fluid retention and (C) without any anastomotic complications

There was no preoperative nerve paralysis; however, the patient developed paraplegia on postoperative day one. Cerebrospinal fluid drainage was performed, but there was no improvement in the patient's symptoms. Long‐term (8 weeks) carbapenem antibiotics were employed to treat the AmpC‐producing *C*. *freundii*; however, the patient consequently developed pseudomembranous enteritis caused by *Clostridioides difficile*. Pseudomembranous enteritis was diagnosed by colonoscopy. Vancomycin and Fidaxomicin were used to treat *C*. *difficile*, and the patient was discharged on postoperative day 255. The blood culture at discharge was negative, and blood results also improved (white blood cell count of 72×10^2^/μL and C‐reactive protein level of 3.3 mg/dL). A CT scan performed at the time of discharge showed neither anastomotic complications nor retroperitoneal fluid retention (Figure 3). However, the paraplegia persisted. Voluntary movements of the lower body were not possible, and sensory impairment remained.

## 3 DISCUSSION

The morbidity and mortality rates of infectious aortic aneurysm rupture are extremely high, with a poor prognosis for non‐operative cases.^6^ The causes of infectious aneurysms are as follows: bacterial aneurysms caused by an infectious embolus due to endocarditis; arteritis followed by an aneurysm due to bacterial invasion of an arteriosclerotic blood vessel wall; infection of a pre‐existing aneurysm; and traumatic, infectious pseudoaneurysms including those caused iatrogenically.^7^ Our patient had arteriosclerosis and a chronic dissecting lesion, which served as a nidus of infection. In the absence of a pre‐existing aneurysm, the patient was diagnosed with arteritis‐type aneurysm formation.

*Salmonella* is the most common causative organism for this type of infection, followed by *Staphylococci* and *Streptococci*.^8^ In the present case, AmpC‐producing *C*. *freundii* was the causative organism and required long‐term (8 weeks) administration of carbapenem antibiotics. The antibiotics caused pseudomembranous enteritis due to *C*. *difficile*, which prolonged the duration of treatment. We administered vancomycin (10 days) and fidaxomicin (14 days) to eradicate *C*. *difficile*; fidaxomicin was particularly effective in the patient.

*C*. *freundii* is mostly found in patients who are immunosuppressed; however, our patient's immune status was normal. A mortality rate of 6.8% has been reported among hospitalized patients with *Citrobacter* infections, but it can significantly increase to 17.8%–56% in those with *Citrobacter* bacteremia.^9^ Multidrug‐resistant *C*. *freundii* strains have been associated with a higher rate of in‐hospital mortality rates compared to susceptible strains.^10^

Prevention of infections in prosthetic aortic grafts is important in the overall treatment strategy, and the following methods should be considered: 1) meticulous removal of the abscess; 2) muscle and omental flap wrapping^11^; 3) addition of antibacterial agents to the prosthetic grafts (eg, rifampicin soaking)^12^; 4) the use of a homograft^13^; and 5) extra‐anatomical revascularization.^1^ In the present case, the patient's condition led to an emergency situation, and it was impractical to obtain a homograft. Extra‐anatomical reconstruction was also non‐viable for a TAAA requiring reconstruction of the major abdominal branch. Therefore, methods 1–3 were used to avoid infection of the grafts. If complete removal of the arterial wall containing the infected tissue is intended in infectious TAAA, reconstruction of the intercostal artery is not viable, and spinal cord ischemia is a concern. In this case, widespread infection made it difficult to reconstruct the intercostal artery and consequently led to paraplegia. A review article showed that in the acute presentation of thoracoabdominal aortic disease, endovascular surgery has evolved as a viable alternative therapy or as a bridge to a more definitive repair.^14^ However, in this case, infection and rupture occurred at the same time, and open surgery was still the first choice.

## 4 CONCLUSION

We encountered a rare case of infectious TAAA due to *C*. *freundii* invasion of a pre‐existing dissecting aortic lesion, which led to aortic rupture within a week. AmpC production by the organism required administration of carbapenem antibiotics that resulted in pseudomembranous enteritis. The findings suggest that *C*. *freundii*, an opportunistic bacterium, can infect residual dissections in the descending aorta, and the resulting infectious aortic aneurysm could rupture in a week. A rifampicin‐soaked prosthesis and omental flap wrapping may be effective in preventing vascular prosthesis infection.

## CONFLICT OF INTEREST

The authors have no conflict of interest to declare.

## AUTHOR CONTRIBUTIONS

Masafumi Hashimoto served as a corresponding author. Kenji Mogi and Manabu Sakurai approved the manuscript. Tomoki Sakata drafted the manuscript. Kengo Tani collected the data. Yoshiharu Takahara involved in critical revision of the manuscript.

## Data Availability

The data that support the findings of this study are available on request from the corresponding author. The data are not publicly available due to privacy or ethical restrictions.
